# Household relationships and healthcare seeking behaviour for common childhood illnesses in sub-Saharan Africa: a cross-national mixed effects analysis

**DOI:** 10.1186/s12913-019-4142-x

**Published:** 2019-05-14

**Authors:** Joshua O. Akinyemi, Pamela Banda, Nicole De Wet, Adenike E. Akosile, Clifford O. Odimegwu

**Affiliations:** 1 Demography and Population Studies Programme, Schools of Public Health and Social Sciences, University of the Witwatersrand, Johannesburg, South Africa; 2 Department of Epidemiology and Medical Statistics, Faculty of Public Health, College of Medicine, University of Ibadan, Ibadan, Nigeria; 3 Department of Sociology, Covenant University, Ota, Nigeria

**Keywords:** Family context, Household relationships, Childhood illnesses, Healthcare seeking behaviour, Healthcare utilisation, Sub-Saharan Africa

## Abstract

**Background:**

Intra-household dynamics play crucial roles in utilisation of healthcare services for children. We investigated the influence of household relationships on healthcare seeking behaviour for common childhood illnesses in four sub-Sahara African regions.

**Methods:**

Data on 247,061 under-five children were extracted from recent Demographic and Health Surveys conducted between 2012 and 2016 in 25 countries. Data were combined and analysed per sub-region. Dependent variables (DVs) were uptake of health facility care for diarrhea and Acute Respiratory Tract Infection (ARI) symptoms. The main independent variable (IV) was household relationship which was represented by maternal marital profile (marital status, family type and number of marriages) and maternal relationship to household head. Mixed effects logit models were fitted to assess independent relationship between the IVs and DVs with adjustment for relevant demographic and socio-economic characteristics at 5% significance level.

**Results:**

The percentage of children who received care for diarrhea and ARI symptoms from health facilities across sub-regions was: Western Africa (WA) 42.4, 44.1%; Central Africa (CA) 32.6, 33.9%; Eastern Africa (EA) 41.5, 48.7% and Southern Africa (SA) 58.9, 62.7%. Maternal marital profile was not associated with healthcare seeking behaviour for diarrhea and ARI symptoms in any of the sub-regions. Children whose mothers were daughter/daughter-in-law to household head were significantly less likely to be taken to health facility for diarrhea treatment in Eastern Africa (AOR = 0.81, CI: 0.51–0.95). Having a mother who is the head of household was significantly associated with higher odds of facility care for ARI symptoms for children from Western (AOR = 1.20, CI: 1.02–1.43) and Southern Africa (AOR = 1.49, CI: 1.20–1.85).

**Conclusion:**

The type of relationship between mother of under-fives and head of households affect health seeking behaviour for treatment of diarrhea and ARI symptoms in Eastern, Western and Southern Africa. Countries in these regions need to adapt best practices for promoting healthcare utilisation for children such that household relationship does not constitute barriers.

**Electronic supplementary material:**

The online version of this article (10.1186/s12913-019-4142-x) contains supplementary material, which is available to authorized users.

## Background

Though there has been steady progress in reduction of childhood deaths from infectious causes, the magnitude is still substantial in sub-Saharan Africa region. Approximately, 50% of global under-five deaths were from the region [1]. Globally, half of childhood deaths are due to infections - with pneumonia, diarrhea and measles leading the trail [1, 2]. Fortunately, these conditions are both preventable and treatable. The recommended point of care and treatment for childhood illnesses is health facility [3]. A persistent challenge in sub-Saharan Africa and many other developing regions is poor healthcare seeking behaviour despite availability of effective treatments for childhood diseases. According to findings from a systematic review, care-seeking from community health workers for diarrhoea, pneumonia and malaria was very low in developing countries [3].

Meanwhile, the second target under the third Sustainable Development Goal (SDG 3) is aimed at ending preventable neonatal and under-five deaths by the year 2030. For this goal to be achieved, efforts need to be geared towards improving healthcare seeking behaviour for childhood illnesses. In poor resource settings such as those found in many sub-Saharan Africa communities, household relationships constitute a challenge to healthcare utilisation. Household relationships are not static; they are subject to the prevailing socio-economic and development ecosystem in different countries [4–6]. Similarly, the effect of household relationships on healthcare seeking behaviour is transient in nature, thereby necessitating occasional re-appraisal [6–8].

Literature has shown that childhood mortality, morbidity, and healthcare seeking behaviour share similarities in terms of associated factors [9–12] in which socio-economic and demographic variables are prominent. Women with secondary or higher education and those living in rich households are more likely to seek appropriate care when their children are ill compared to those with no formal education and those from poor households [13–15], respectively. Likewise, urban dwellers seek appropriate care more than those in rural areas [16, 17]. Meanwhile, demographic variables often found related to healthcare access include age of child and number of under-five children in the household. Children aged below 2 years enjoyed better care-seeking [14, 15, 18] while the number of under-fives in the household is negatively associated with healthcare seeking behaviour [16, 19]. Decision-making power, access to and control over economic resources by mothers of under-five children have also been reported to positively influence care-seeking for childhood illnesses [20, 21]. Distance to health facilities and inability to raise money to pay for cost of treatment are other barriers that have been identified by past studies [18, 19, 22]. Beyond the aforementioned factors, another important social determinant of children health is the family/household environment which includes features such as family structure, social support and intra-household relationships [23]. With research suggesting that access to health is multidimensional [24], analytical studies are needed to investigate other variables such as social structure, family and community characteristics.

Some previous studies, mostly qualitative in design, have shown evidence to underscore the importance of the family environment in care-seeking for childhood illnesses [25–28]. Two main revelations emerged from these qualitative studies. First, decision to seek appropriate health care for a sick child depends on the household’s social context, social structure, social networks and support within the family [21, 27–29]. Second, there is existence of relationship dynamics especially between a mother and the household head. Father’s presence or absence and/or his disposition to the mother determine whether or not a sick child is taken to health facility for treatment [21, 25, 27, 30]. The influence of these intra-household dynamics needs to be investigated on a larger scale.

In this study, we explored these issues using available nationally representative survey data in sub-Saharan Africa, bearing in mind the diversities across different sub-regions. Further investigation of factors affecting care-seeking behaviour for childhood illnesses is imperative for two reasons. First, the roles of families and communities remain central as seen in the strategic revision to the World Health Organisation’s (WHO) guidelines on Integrated Management of Neonatal and Childhood Illnesses [31]. Two, the topmost research priority identified as having potential to reduce childhood deaths from pneumonia (treatable but a leading cause of childhood mortality) is investigation of barriers to healthcare access [32]. In view of these imperatives, we investigated the influence of household relationship on healthcare seeking behaviour for childhood illnesses in sub-Saharan Africa. Of the three common childhood illnesses, the topmost two - diarrhea and ARI symptoms were selected. Besides, information about them is readily available from household surveys. We hypothesized that the relationship between mother of under-five and the household head affects care-seeking behaviour for diarrhea and ARI symptoms.

## Methods

This study involved secondary analysis of cross-sectional data extracted from the most recent Demographic and Health Surveys (DHS) conducted in 25 sub-Saharan African countries between 2012 and 2016 (Additional file 1: Table S1). The DHS data are nationally representative household surveys conducted in several developing countries since the late 1990s [33]. The surveys cover several topics related to population health of which maternal and child health is prominent. Men and women (both aged 15–59 years old) are usually selected for interview via stratified two-stage cluster sampling technique. The primary sampling units are census enumeration areas (EAs). These EAs are stratified by rural and urban localities. The first stage of sampling is selection of specified number of EAs in rural and urban localities. In selected EAs, household listing and numbering is done to generate sampling frame for second stage of sampling. Depending on the sample size required, a fixed number of households are subsequently selected using a systematic sampling with probability proportional to size (number of households) of the EAs. Variables and data collection methodologies are standardized across countries and surveys thus facilitating multi-country analyses [34]. Data on all children ever born to women were collected in the reproductive history section of the women’s questionnaire. Additional data on health and anthropometry characteristics of children alive at the time of survey were also collected. Data were collected using standardized questionnaire by trained field workers. In this study, we analysed data for a weighted sample of 247,061 children who were alive during the surveys. Though the data analysed covered an average of 5-year period, this time interval is narrow and did not affect the overall findings of the study.

### Theoretical framework

For theoretical foundation, we adapted the phase 5 model of health services use [35]. The model which was first proposed in the late 1960s has been applied in several studies of healthcare utilisation and has been reviewed five times to capture the complex nature of factors related to health-seeking behaviour [35, 36]. In the most recent version, environmental or contextual factors and population characteristics were argued to influence the use of health services. Environmental factors include healthcare system, resources, social and economic development. Population characteristics were represented by demographics, family social structure and community characteristics. In this study, a sub-component of the relationships hypothesized in the model is empirically investigated by focusing on some components under population characteristics and their effect on healthcare seeking behaviour for childhood illnesses.

### Variables

Two dependent variables were analysed and these are healthcare seeking behaviour for diarrhea and Acute Respiratory Tract Infection (ARI) symptoms. The variables were dichotomous, coded Good [1] and Poor (0). In this study, good healthcare seeking behaviour refers to public or private health facility visit to access care for diarrhea or ARI symptoms. Care-seeking for diarrhea was derived from three set of questions under the child immunisation, health and nutrition section of the DHS women’s questionnaire. The first question was “Has child had diarrhea in the last 2 weeks”. If mother answered “Yes”, she was asked the second question – “Did you seek advice or treatment for the diarrhea from any source”. With an affirmative response to the second question, the interviewer proceeded to the third one – “where did you seek advice or treatment”.

Care-seeking for ARI symptoms was derived in similar manner to diarrhea. The first question posed to a mother was “Has child had an illness with a cough at any time in the last 2 weeks?” If mother answered ‘Yes’, she was further asked “When child had an illness with a cough, did he/she breath faster than usual with short, rapid breaths or have difficulty breathing?”. Children for whom the answer to these two questions was ‘Yes’ were categorised as having ARI symptoms. The third and fourth question which relate to care-seeking was “Did you seek advice or treatment for the illness from any source?” and “where did you seek advice or treatment?”

Two explanatory variables were employed to represent household relationships. These were maternal marital profile and relationship to household head. Maternal marital profile has six categories that captured the marital status, family type and number of marriages a mother has had. The categories were: 1- never married; 2- first order monogamy, 3- first order polygyny, 4- higher order monogamy, 5- higher order polygyny and 6- formerly married (separated, divorced or widowed). Order of a marriage is the number of times a woman has been married. Therefore, first order means that a woman has been married only once while higher order implied being married two or more times. Monogamy is the situation whereby a woman report that her partner do not have other wives while polygyny implied that there are other co-wives. Relationship between the mother of under-five and household head was categorised as 1-head of household (that is the mother is also the household head), 2- wife, 3- daughter or daughter-in-law and 4- others.

Based on existing literature, we adjusted for other background variables representing the demographic and socio-economic characteristics of the mother and household [14, 16, 37]. These were maternal education, maternal age, occupation, household wealth index, place of residence, sex of child, age of child, birth order, and number of under-five children in the household.

### Statistical analysis

Analysis was done for sub-Saharan Africa as a whole and for each sub-region [Western Africa; Central Africa; Eastern Africa; Southern Africa]. To provide a description of the study variables, weighted percentage distributions were estimated. In order to account for the complex sampling scheme employed in the DHSs and the hierarchical structure of the data, three-level mixed effects logit models were fitted to explore the independent association between household relationships and healthcare seeking behaviour for diarrhea and ARI symptoms. In this study, children-mother pairs were nested in clusters or communities (primary sampling unit) which were in turn nested within countries. Thus, for the three-level mixed effects model, mother-child pairs represents level 1, clusters are level 2, while countries represent level 3. Estimation models for each sub-region were based on the equation below:

$$ {Log}_e\left[\frac{p_{ijk}}{1-{p}_{ijk}}\right]={X}_{ijk}+{\mu}_{jk}+{v}_k $$

Where:

*P*_*ijk*_= probability of good healthcare seeking behaviour by mother-child pair *i*, in community *j* and country *k.*

*X*_*ijk*_ = vector of explanatory variables.

β = regression coefficient representing effects of respective explanatory variable on probability of good health seeking behaviour.

μ_jk_= community-level variance.

*v*_*k*_ = country-level variance.

Models were fitted in two stages. In the first model, each of the variables for household relationship was used to estimate unadjusted effects. Thereafter, to obtain adjusted effects, demographic and socio-economic variables were added to the model. We did not use any variable selection technique because we were interested in an explanatory rather than a predictive model. The explanatory model was aimed at clarifying the independent association between household relationship and healthcare seeking behaviour.

Mixed effects models have two components – random and fixed. Random effects were estimated at cluster (community) and country levels. Fixed effects of explanatory variables were estimated as Adjusted Odds Ratio (AOR) with 95% Confidence Interval (CI). Model fitness was assessed and all anayses were conducted using Stata MP version 14.

## Results

### Healthcare seeking behaviour for diarrhea

Of a weighted sample of 247,061 under-five children, 36,865 (14.9%) were reported to have had diarrhea and 23,135 (9.4%), ARI symptom. Healthcare seeking behaviour is presented in Table 1 with left panel for diarrhea and right panel for ARI symptoms. By region, the percentage of children whose mothers sought treatment for diarrhea from health facility was: Western Africa (WA)– 42.4%, Central Africa (CA) – 32.6%, Eastern Africa (EA) – 41.5% and Southern Africa (SA) – 58.9%. Health facility care for diarrhea was almost evenly distributed according to maternal age except for those aged 40 years and above who were slightly lower than the other age groups. Educational gaps were observed in WA, CA, and EA where facility care for diarrhea was more common in children whose mothers had secondary/higher education than those with no formal education. In WA, 35.3 and 47.9% of under-five children in poorest and richest household respectively received facility care for diarrhea, while 27.7 and 38.8% respectively for CA. There were no such differentials in EA and SA sub-regions. Similarly, seeking of health facility care for diarrhea was slightly more common in urban compared to rural areas in WA and CA, unlike the other two sub-regions where no such disparity was observed.

**Table 1:** Demographic and socio-economic characteristics of mothers of under-five children who sought care for diarrhea and ARI symptoms from health facilities in sub-Saharan Africa, 2012–2016

	Health facility care for diarrhea					Health facility care for ARI symptoms				
	Western Africa	Central Africa	Eastern Africa	Southern Africa	sub-Saharan Africa	Western Africa	Central Africa	Eastern Africa	Southern Africa	sub-Saharan Africa
Maternal age (years)										
< 20	42.0	35.6	37.5	60.7	44.7	46.7	35.9	46.7	63.2	47.2
20–29	42.6	32.5	42.2	59.2	43.8	44.0	34.5	50.1	63.5	46.8
30–39	43.0	32.9	42.3	57.6	43.3	44.2	33.4	48.2	61.6	45.7
> =40	39.2	27.6	37.2	58.7	39.3	42.6	30.0	42.5	60.3	42.4
Maternal education										
None	40.5	25.3	39.7	53.8	38.0	41.3	22.4	39.2	62.2	37.9
Primary	42.2	33.3	39.8	60.9	45.7	42.0	33.3	46.9	61.8	46.9
Secondary/higher	48.8	38.5	47.4	57.0	47.8	54.0	44.7	57.3	64.0	54.5
Employment status										
Working	41.6	33.7	33.6	60.3	42.3	44.2	34.6	42.8	66.1	45.6
Not working	43.7	31.2	40.8	56.9	43.4	44.0	33.0	48.2	58.1	44.9
Household wealth quintile										
Poorest	35.3	27.7	44.9	57.9	40.0	37.3	26.6	46.6	61.1	41.6
Poor	41.5	31.9	42.0	58.1	42.9	39.5	32.7	50.3	59.5	44.1
Middle	43.8	32.3	41.5	61.2	44.2	45.5	32.6	44.9	64.9	45.9
Richer	46.7	34.0	39.4	60.1	45.4	48.2	36.1	48.7	63.5	48.2
Richest	47.9	38.8	37.6	57.2	45.9	52.9	45.2	54.8	66.2	53.6
Residence										
Rural	40.7	31.2	41.5	59.6	43.1	41.0	28.5	48.2	62.2	44.3
Urban	45.9	34.8	41.6	57.0	44.1	50.6	43.2	49.9	64.1	50.3
Age of child (months)										
0–11	43.2	34.0	42.2	57.1	43.5	45.8	36.0	49.3	63.7	47.1
12–23	45.7	32.6	40.8	62.9	45.8	46.2	39.5	50.1	64.2	48.8
24–35	40.6	32.2	41.9	58.7	42.5	43.8	31.3	47.7	63.5	45.6
36–59	38.9	31.4	41.6	54.4	40.6	40.9	29.5	47.9	60.2	43.6
Birth order										
1	43.3	36.5	39.3	60.7	45.8	47.2	42.2	49.5	65.5	50.8
2–3	43.5	32.2	44.0	57.6	44.3	45.5	34.7	52.5	62.4	48.3
4–5	42.2	32.4	40.8	59.4	43.1	43.1	31.1	46.7	61.1	44.0
> =6	40.1	30.3	40.0	57.8	39.6	40.1	29.0	40.6	60.4	39.4
No of under-5 in household										
< = 2	42.9	34.2	42.6	59.1	45.0	44.3	36.4	49.5	64.1	48.2
> =3	41.6	29.2	37.0	57.7	39.4	43.8	29.0	45.2	53.3	40.4

In terms of household relationship (Table 2, left panel), children of women in polygynous households recorded poorer visit of health facility for diarrhea treatment in WA and CA whereas first and higher order monogamy had the poorest uptake in EA. For SA sub-region, no major differential was observed; however children of never married women (63.0%) had the highest utilisation of health facility while higher order polygyny and first order monogamy had 56.2 and 59.2% respectively. Children of women who were wives to head of household (40.1%) had the lowest utilisation of health facility for diarrhea treatment in WA whereas the lowest in CA was among children whose mothers were household heads (30.7%) and among children whose mothers were daughter or daughter-in-law to household head in EA.

**Table 2:** Household relationships among mothers of under-five children who sought care for diarrhea and ARI symptoms from health facilities in sub-Saharan Africa, 2012–2016

	Health facility care for diarrhea					Health facility care for ARI symptoms				
Variables	Western Africa	Central Africa	Eastern Africa	Southern Africa	sub-Saharan Africa	Western Africa	Central Africa	Eastern Africa	Southern Africa	sub-Saharan Africa
Maternal marital profile										
Never married	46.2	40	43.2	63	50.1	48.2	51	55.4	64.3	55.3
First order marriage, monogamy	42.7	33.2	36.7	59.2	43.4	44.7	34.8	45.7	63.3	46.3
First order marriage, polygyny	40.6	28.6	51.4	56.9	41.2	42.2	29.9	53.3	59.3	44.4
Higher order marriage, monogamy	45.1	37.8	31.9	55.8	44.7	47.0	36.5	30.3	60.6	45.5
Higher order marriage, polygyny	39.2	29.2	41.6	56.2	39.9	39	27.2	50.8	55.6	40.3
Formerly married	44.4	31.4	41.7	58.6	44.3	44.4	28.7	50.1	64.7	46.9
Maternal relationship to household head										
Head	46.5	30.7	48.1	58.6	46.8	50.9	34.3	50.4	65.6	50.5
Wife	40.1	32.6	41.7	58.5	42	41.7	33.6	48.2	61.3	44.4
Daughter/in-law	47.2	32.5	35.4	60.3	45.5	46.7	34.2	48.7	61	47.5
Others	48.7	36.2	34.5	59.8	46.7	50.2	36.8	49.1	71.5	51.7

### Healthcare seeking behaviour for ARI symptoms

Among children with ARI symptoms, the percentage for whom care was sought from health facility across the four sub-regions was: WA – 44.1%; CA – 33.9%; EA – 48.7%; SA – 62.7% (Table 1, right panel). The percentage did not vary by maternal age but educational differentials were observed in WA, CA and EA where children whose mothers had secondary/higher education had the highest rates of health facility utilisation. Table 1 (right panel) also showed that health facility care-seeking for ARI symptoms was more common among children with working (66.1%) than non-working mother (58.1%) in SA. The widest wealth differential was observed in WA between the poorest (37.3%) and richest households (52.9%) which was closely followed by CA (poorest – 26.6%, richest – 45.2%) and EA (poorest – 46.6%, richest – 54.8%). Care-seeking for ARI symptoms in SA was very close between the poorest (61.1%) and richest (66.2%) households. The percentage for whom healthcare was sought from health facility was lower among children in rural areas in WA (rural – 41%, urban – 50.6%) and CA (rural – 28.5%, urban – 43.2%). Rural-urban differential was nearly absent in EA and SA.

With respect to household relationship, Table 2 (right panel) showed that children of women in higher order polygynous unions recorded the poorest utilisation of health facility for ARI symptoms in WA (39.0%) and CA (27.2%). Again, health facility care-seeking was lowest among children whose mothers were wives (41.7%) to household head in WA and highest among those with mothers as household heads (50.9%).

### Multivariable analyses

Results from models fitted to investigate the independent effect of household relationship on healthcare seeking behaviour are presented in Table 3 (diarrhea) and Table 4 (ARI symptoms). Even though, explanatory models which include all variables were fitted, we present results for only household relationship which is the primary focus of this study. Odds Ratio from unadjusted and adjusted models was quite similar. Table 3 (panel 2) shows that most of the variables did not attain statistical significance in their association with healthcare seeking behaviour for childhood diarrhea. Higher order monogamy was associated with lower odds of health facility care for diarrhea in Western Africa (OR = 0.89, CI: 0.78–1.03). In Eastern Africa, mothers who never married (OR = 1.43, CI: 0.97–2.11) and those in first order polygynous unions (OR = 1.19, CI: 0.89–1.57) were more likely to seek health facility care for childhood diarrhea. Compared to housewives, daughters/daughters-in-laws were significantly less likely of taking their children to health facility for appropriate diarrhea treatment (OR = 0.86, CI: 0.51–0.95).

**Table 3:** Unadjusted and adjusted effect of household relationships on health seeking behaviour for childhood diarrhea in sub-Saharan Africa, 2012–2016

	Unadjusted Odds Ratio (OR)				
Caregiver characteristics	Western Africa	Middle Africa	Eastern Africa	Southern Africa	sub-Saharan Africa
Marital profile					
Never married	0.99 (0.84–1.18)	0.98 (0.78–1.25)	1.17 (0.86–1.60)	1.02 (0.83–1.26)	1.01 (0.91–1.13)
First order marriage, monogamy	1.00	1.00	1.00	1.00	1.00
First order marriage, polygyny	0.94 (0.86–1.02)	0.87 (0.77–1.00)	0.98 (0.84–1.15)	0.86 (0.71–1.05)	0.92 (0.87–0.98)*
Higher order marriage, monogamy	0.88 (0.77–1.01)	1.08 (0.91–1.27)	0.99 (0.74–1.33)	0.85 (0.72–1.00)	0.92 (0.8401.01)
Higher order marriage, polygyny	0.89 (0.77–1.04)	0.94 (0.76–1.17)	0.82 (0.60–1.11)	0.78 (0.60–1.02)	0.88 (0.79–0.97)*
Formerly married	0.88 (0.73–1.06)	0.93 (0.75–1.14)	1.09 (0.85–1.42)	0.90 (0.75–1.09)	0.92 (0.84–1.02)
Relationship to household head					
Head	1.04 (0.92–1.19)	0.92 (0.78–1.09)	0.88 (0.74–1.05)	1.08 (0.92–1.26)	0.99 (0.92–1.07)
Wife	1.00	1.00	1.00	1.00	1.00
Daughter/in-law	1.10 (0.99–1.23)	0.99 (0.78–1.09)	0.80 (0.63–1.01)	1.06 (0.89–1.25)	1.03 (0.95–1.11)
Others	1.02 (0.89–1.16)	1.03 (0.83–1.27)	0.76 (0.53–1.09)	1.10 (0.87–1.38)	1.00 (0.91–1.11)
	Adjusted OR^++^				
Caregiver marital profile					
Never married	0.97 (0.81–1.15)	0.97 (0.76–1.23)	1.43 (0.97–2.11)	1.00 (0.81–1.24)	1.00 (0.89–1.12)
First order marriage, monogamy	1.00	1.00	1.00	1.00	1.00
First order marriage, polygyny	0.99 (0.91–1.09)	0.92 (0.80–1.05)	1.19 (0.89–1.57)	0.88 (0.73–1.07)	0.98 (0.91–1.04)
Higher order marriage, monogamy	0.89 (0.78–1.03)	1.09 (0.92–1.30)	1.05 (0.78–1.42)	0.88 (0.74–1.05)	0.95 (0.87–1.04)
Higher order marriage, polygyny	0.95 (0.81–1.09)	0.98 (0.79–1.22)	0.82 (0.49–1.39)	0.83 (0.64–1.09)	0.94 (0.84–1.04)
Formerly married	0.90 (0.75–1.09)	0.95 (0.78–1.18)	1.11 (0.80–1.52)	0.93 (0.76–1.13)	0.95 (0.86–1.06)
Relationship to household head					
Head	1.03 (0.90–1.17)	0.93 (0.79–1.10)	0.81 (0.65–1.00)	1.08 (0.92–1.27)	0.99 (0.92–1.08)
Wife	1.00	1.00	1.00	1.00	1.00
Daughter/in-law	1.06 (0.95–1.19)	0.97 (0.81–1.15)	0.81 (0.51–0.95)*	1.04 (0.84–1.24)	0.99 (0.92–1.08)
Others	0.99 (0.86–1.14)	1.02 (0.82–1.27)	0.90 (0.86–1.71)	1.06 (0.84–1.36)	0.99 (0.90–1.10)
Random effects parameters					
Country: Variance (SE)	0.3789 (0.1613)	0.0229 (0.0234)	15.6738 (14.5082)	0.1421 (0.0964)	1.6615 (0.5506)
Community: Variance (SE)	0.1765 (0.0278)	0.2043 (0.0416)	0.0647 (0.0386)	0.0876 (0.0235)	0.1507 (0.0164)
Model parameters					
Log-likelihood	− 9717.6826	− 5193.6512	− 1895.0743	− 4858.7665	− 2176.202
Wald statistic	175.33	95.04	36.16	79.28	269.88
*p*-value	< 0.001	< 0.001	0.0888	< 0.001	< 0.001

*p < 0.05 based on models adjusted for demographic and socio-economic characteristics

**Table 4:** Unadjusted and adjusted effect of household relationships on health seeking behaviour for ARI symptoms among under-fives in sub-Saharan Africa, 2012–2016

	Unadjusted OR				
Caregiver characteristics	Western Africa	Middle Africa	Eastern Africa	Southern Africa	sub-Saharan Africa
Marital profile					
Never married	1.02 (0.81–1.29)	1.19 (0.89–1.59)	1.15 (0.84–1.58)	0.91 (0.68–1.20)	1.09 (0.95–1.25)
First order marriage, monogamy	1.00	1.00	1.00	1.00	1.00
First order marriage, polygyny	0.96 (0.85–1.08)	0.86 (0.73–1.02)	0.87 (0.75–1.02)	0.88 (0.69–1.13)	0.90 (0.84–0.97)*
Higher order marriage, monogamy	0.88 (0.74–1.04)	0.79 (0.64–0.97)*	0.89 (0.62–1.27)	0.84 (0.67–1.06)	0.85 (0.76–0.95)*
Higher order marriage, polygyny	0.82 (0.68–0.99)*	0.79 (0.60–1.02)	1.04 (0.76–1.44)	0.65 (0.46–0.91)*	0.82 (0.72–0.93)*
Formerly married	0.81 (0.63–1.04)	0.79 (0.61–1.02)	1.02 (0.79–1.33)	0.84 (0.64–1.09)	0.89 (0.78–1.00)
Relationship to household head					
Head	1.04 (0.92–1.19)	0.92 (0.78–1.09)	0.88 (0.74–1.05)	1.08 (0.92–1.26)	1.13 (1.03–1.24)*
Wife	1.00	1.00	1.00	1.00	1.00
Daughter/in-law	1.10 (0.99–1.23)	0.99 (0.84–1.17)	0.80 (0.63–1.01)	1.06 (0.90–1.25)	1.02 (0.93–1.12)
Others	1.02 (0.89–1.16)	1.03 (0.83–1.27)	0.76 (0.53–1.09)	1.10 (0.87–1.38)	1.17 (1.03–1.33)*
	Adjusted OR^++^				
Caregiver marital profile					
Never married	0.92 (0.72–1.17)	1.14 (0.85–1.54)	1.07 (0.71–1.61)	0.86 (0.65–1.15)	1.01 (0.88–1.17)
First order marriage, monogamy	1.00	1.00	1.00	1.00	1.00
First order marriage, polygyny	1.00 (0.88–113)	0.92 (0.77–1.09)	0.79 (0.58–1.09)	0.94 (0.73–1.21)	0.98 (0.89–1.07)
Higher order marriage, monogamy	0.92 (0.78–1.10)	0.82 (0.66–1.01)	0.97 (0.67–1.41)	0.90 (0.71–1.14)	0.89 (0.80–1.00)
Higher order marriage, polygyny	0.86 (0.71 (1.05)	0.82 (0.63–1.07)	1.12 (0.66–1.89)	0.71 (0.50–1.00)	0.85 (0.74–0.98)*
Formerly married	0.83 (0.65–1.07)	0.82 (0.63–1.06)	1.00 (0.71–1.40)	0.86 (0.66–1.13)	0.91 (0.79–1.04)
Relationship to household head					
Head	1.20 (1.02–1.43)*	1.04 (0.85–1.27)	1.04 (0.82–1.32)	1.49 (1.20–1.85)*	1.17 (1.06–1.29)
Wife	1.00	1.00	1.00	1.00	1.00
Daughter/in-law	0.94 (0.81–1.09)	0.92 (0.74–1.13)	0.94 (0.67–1.31)	1.17 (0.93–1.49)	0.95 (0.86–1.05)
Others	0.99 (0.82–1.18)	0.97 (0.72–1.29)	1.62 (0.96–2.74)	1.89 (1.35–2.66)*	1.11 (0.97–1.27)
Random effects parameters					
Country: Variance (SE)	0.2739 (0.1219)	0.06305 (0.05523)	13.8080 (12.8859)	0.1689 (0.1143)	1.3434 (0.4517)
Community: Variance (SE)	0.2821 (0.0502)	0.17749 (0.0460)	0.1781 (0.0551)	0.0569 (0.0247)	0.1861 (0.0231)
Model parameters					
Log-likelihood	− 5476.2264	− 3326.2409	− 1767.8458	− 2651.6569	−13,310.475
Wald statistic	148.32	132.1	67.46	88.94	324.54
p-value	< 0.001	< 0.001	< 0.001	< 0.001	< 0.001

*p < 0.05; ++ based on models adjusted for demographic and socio-economic characteristics

Results for ARI symptoms (Table 4, panel 2) shows that formerly married women in WA (AOR = 0.83, CI: 0.65–1.07), CA (AOR = 0.82, CI: 0.63–1.06) and SA (AOR = 0.86, CI: 0.66–1.13) were less likely of seeking healthcare for child ARI symptoms from health facility compared to women in first order monogamous marriages. In addition, higher order polygyny was associated with lower odds of health facility care for ARI symptoms in WA (AOR = 0.86, CI: 0.71–1.05), CA (AOR = 0.82, CI: 0.63–1.07) and SA (AOR = 0.71, CI: 0.50–1.00). Having a mother who is the head of household was significantly associated with higher odds of facility care for ARI symptoms in children from Western Africa (AOR = 1.20, CI: 1.02–1.43) and Southern Africa (AOR = 1.49, CI: 1.20–1.85).

## Discussion

We explored the influence of household relationships on healthcare seeking behaviour for diarrhea and symptoms of ARI – two leading childhood illnesses in sub-Saharan Africa regions. Our results showed that less than half of mothers of children affected by diarrhea and ARI symptoms sought care from health facility. These levels of healthcare utilisation represent some improvement when compared to the situation about a decade ago [38]. There are different pathways to care-seeking for childhood illnesses by mothers of under-fives in the region. In many sub-Saharan Africa settings, the common practice is home management and spiritual / traditional healers before health facility treatment [26, 30]. This healthcare seeking behaviour is often linked to perceptions about causes of illnesses and source(s) of solution [15, 39]. When there is a belief that illness in a child is caused by some spiritual forces or due to violation of certain traditional norms, the parents are more likely to seek healthcare from a traditional healer who they believe knows how to appease the gods [40]. Lowest uptake of health facility care for diarrhea and ARI symptoms in Central Africa possibly explains why the sub-region ranks high in childhood mortality in sub-Saharan Africa. Congo DR, the largest country in the sub-region is recovering gradually from a conflict situation. Communities ravaged by internal conflicts and displacements suffer enormous deterioration in terms of health facilities [41, 42].

Furthermore, comparison of unadjusted and adjusted measures of association between household relationship and healthcare seeking behaviour showed very little differences. This implies that demographic and socio-economic factors do not necessarily explain the influence of household relationships. The roles of intra-household relationship in healthcare utilisation are not mediated by prevailing socio-economic status. The finding implied that household social environment cannot be subsumed under socio-economic factors as far as child healthcare utilisation is concerned.

Although not statistically significant, household relationships showed stronger association with care-seeking behaviour for treatment of ARI symptoms than diarrhea. This is similar to evidence from an assessment of child health inequality conducted by the WHO which revealed that the magnitude of inequality in treatment of ARI symptoms was greater than that of diarrhea [38]. Though it is nearly impossible to unravel the reasons for these differences using the data analysed, it suggests that the effect of household relationship on care-seeking behaviour depend on the specific health condition. Perception of illness causes and navigation of treatment options have been suggested to vary across household and communities [27].

Having a mother who is daughter/daughter-in-law to head of household was associated with lesser chances of health facility care for childhood diarrhea in Eastern Africa. Concerning daughters/daughters-in-laws, socio-cultural norms in many sub-Saharan Africa contexts demand that they defer to their parents or parents-in-law in decision-making on child healthcare. Ethnographic data from Northern Ghana actually confirms grandmothers as sources of social support for young mothers [29]. In their roles as guardians, they often favour seeking treatment from traditional healers thereby discouraging the use of health facilities [29].

In regard to care-seeking for ARI symptoms, though the direction of relationship is similar across the four sub-regions, the magnitude varied. These differences might be due to variables related to health system which is not uniform across settings. Examples of such variables include child survival interventions implemented by different countries, doctors per population, user fees, and health insurance coverage among others.

Western and Southern Africa mothers who were household heads were significantly more likely of seeking health facility care for ARI symptoms. There are evidences in literature that supports this finding [43, 44]. Mothers of under-five children who also doubled as heads of their households clearly do not experience the problem of getting permission to seek care which is a common barrier to healthcare utilisation for women and children [26, 30, 45]. In addition, such women would most likely not want to take any risk since there is no other person playing the headship role in the household. This finding does not suggest that advocacy be made for mothers of under-five children to become household heads. Rather, it implies that they should be given more freedom to take proactive decisions on child healthcare.

This study has some limitations. Both diarrhea and ARI symptoms were based on maternal reports about the child health condition within two weeks of the interviews. While the problem of recall may be minimal, some women may not have correctly recognised the signs and symptoms of ARI. Inaccurate recognition of ARI symptoms have been reported in some studies [3, 46]. In addition, these health conditions were not based on any medical diagnosis. Therefore, over or under-reporting may not be completely ruled out. Another limitation is the inability to analyse health system/service related factors that may be related to child healthcare seeking behaviour.

One strength of this study is our use of appropriate statistical techniques which accounts for inter country variations. Besides, the study served the purpose of presenting stronger and nationally representative findings to complement evidence from few qualitative studies on intra household dynamics and child healthcare utilisation in SSA.

Our findings have implications for future research and programmes on child health and survival. Further studies are needed to provide innovative strategies on how male household heads can be motivated to proactively support utilisation of healthcare services for children. Apart from women, stronger child health advocacy and awareness targeted at men in Western Africa are needed in view of their roles in household leadership and provision of resources for children healthcare.

## Conclusion

With less than half of affected children receiving care for diarrhea and ARI symptoms from health facilities, care-seeking for treatable causes of childhood death is still below average in sub-Saharan Africa. Though, there are subtle variations in magnitude, the pattern and direction of association with household relationship is consistent across the four sub-regions. Different dimensions of household relationships such as polygyny, higher order marriages, being formerly married and being a daughter or daughter-in-law by a mother of under-five are not associated with healthcare seeking behaviour for childhood diarrhea and ARI symptoms. However, mothers who served as household head in Western and Southern Africa were more likely to seek health facility care for ARI symptoms. Statistical non-significance in the other two sub-regions is an indication that household relationship is not a barrier to care-seeking from health facilities.

## Additional file

Additional file 1: **Table S1.** List of study countries, survey year and weighted sample size. A table showing study countries, survey year and weighted sample size. (DOCX 15 kb)

## Abbreviations

AOR: Adjusted Odds Ratio
ARI: Acute respiratory tract infections
CA: Central Africa
CI: Confidence Interval
DHS: Demographic and Health Survey
DV: Dependent variable
EA: Eastern Africa
IV: Independent variable
OR: Odds Ratio
SA: Southern Africa
SDG: Sustainable Development Goal
SSA: Sub-Saharan Africa
WA: Western Africa
WHO: World Health Organization
