# Supplementary material for: Household relationships and healthcare seeking behaviour for common childhood illnesses in sub-Saharan Africa: a cross-national mixed effects analysis
Source: BMC Health Serv Res. 2019 May 14;19:308. doi: 10.1186/s12913-019-4142-x (PMC6518738; doi:10.1186/s12913-019-4142-x)
Supplement: Supplementary file 1 — Table S1. List of study countries, survey year and weighted sample size. A table showing study countries, survey year and weighted sample size. (DOCX 15 kb) [file 12913_2019_4142_MOESM1_ESM.docx]

**SupplementaryTable S1: List of study countries, survey year and weighted sample size**

| **Sub-region** | **Country** | **Survey year** | **Sample size** |
| --- | --- | --- | --- |
| Western Africa | Benin | 2011/2012 | 12497 |
|  | Cote d'Ivoire | 2011/2012 | 6862 |
|  | Ghana | 2014 | 5431 |
|  | Gambia | 2013 | 7586 |
|  | Guinea | 2012 | 6448 |
|  | Liberia | 2013 | 6047 |
|  | Mali | 2012/2013 | 9655 |
|  | Nigeria | 2013 | 28950 |
|  | Niger | 2012 | 12255 |
|  | Sierra Leone | 2013 | 10814 |
|  | Senegal | 2012/2013 | 6078 |
|  | Togo | 2013/2014 | 6286 |
| Central Africa | Congo DRC | 2013/2014 | 17017 |
|  | Congo Brazzaville | 2011/2012 | 7751 |
|  | Gabon | 2012 | 4848 |
|  | Chad | 2014/2015 | 16810 |
| Eastern Africa | Kenya | 2014 | 18702 |
|  | Comoros | 2012 | 3089 |
|  | Rwanda | 2014 | 7694 |
|  | Tanzania | 2015/2016 | 9520 |
| Southern Africa | Lesotho | 2014 | 2896 |
|  | Malawi | 2015/2016 | 16548 |
|  | Namibia | 2013 | 4588 |
|  | Zambia | 2013 | 12634 |
|  | Zimbabwe | 2015 | 6055 |
